# Increased gene dosage of *Ink4/Arf* and *p53* delays age-associated central nervous system functional decline

**DOI:** 10.1111/acel.12343

**Published:** 2015-05-20

**Authors:** Estefania Carrasco-Garcia, Olatz Arrizabalaga, Manuel Serrano, Robin Lovell-Badge, Ander Matheu

**Affiliations:** 1 Neuro-Oncology Group, Biodonostia InstitutePaseo Dr. Beguiristain s/n, San Sebastian, 20014, Spain; 2 Tumor Suppression Group, Spanish National Cancer Research Centre (CNIO)3 Melchor Fernandez Almagro st, Madrid, 28029, Spain; 3 Francis Crick InstituteMill Hill, London, NW7 1AA, UK

**Keywords:** aging, anti-aging, gerontogenes, Ink4a, neural stem cells, neuroscience, p53, Arf

## Abstract

The impairment of the activity of the brain is a major feature of aging, which coincides with a decrease in the function of neural stem cells. We have previously shown that an extra copy of regulated *Ink4/Arf* and *p53* activity, in s-*Ink4/Arf/p53* mice*,* elongates lifespan and delays aging. In this work, we examined the physiology of the s-*Ink4/Arf/p53* brain with aging, focusing on the neural stem cell (NSC) population. We show that cells derived from old s-*Ink4/Arf/p53* mice display enhanced neurosphere formation and self-renewal activity compared with *wt* controls. This correlates with augmented expression of *Sox2, Sox9, Glast, Ascl1,* and *Ars2* NSC markers in the subventricular zone (SVZ) and in the subgranular zone of the dentate gyrus (DG) niches. Furthermore, aged s-*Ink4/Arf/p53* mice express higher levels of *Doublecortin* and *PSA-NCAM* (neuroblasts) and *NeuN* (neurons) in the olfactory bulbs (OB) and DG, indicating increased neurogenesis *in vivo*. Finally, aged s-*Ink4/Arf/p53* mice present enhanced behavioral and neuromuscular coordination activity. Together, these findings demonstrate that increased but regulated *Ink4/Arf* and *p53* activity ameliorates age-related deterioration of the central nervous system activity required to maintain the stem cell pool, providing a mechanism not only for the extended lifespan but also for the health span of these mice.

The *Ink4/Arf* locus and p53 are regarded as the most relevant tumor suppressors based on their ubiquitous and frequent inactivation in human cancer. In agreement with their damage protection role, enhanced *Ink4/Arf* and p53 activity protects mice from cancer (Garcia-Cao *et al*., 2002; Tyner *et al*., 2002; Maier *et al*., 2004; Matheu *et al*., 2004, 2007, 2009; Mendrysa *et al*., 2006). These mouse models have also revealed that the *Ink4a/Arf* locus and p53 regulate aging-associated pathologies, although the impact of these genes on aging is critically dependent on whether they retain or not their normal regulatory controls. In particular, truncated p53 alleles that permanently activate the endogenous p53 protein display accelerated aging (Tyner *et al*., 2002; Maier *et al*., 2004) and reduced tissue function and regeneration, which suggests a defect in stem cells activity (Dumble *et al*., 2007; Gatza *et al*., 2008; Medrano *et al*., 2009). In contrast, mice carrying an extra transgenic allele of normally regulated p53 or of Ink4/Arf, with Arf being a positive regulator of p53, or with reduced activity of p53’s major negative regulator MDM2, show normal aging (Garcia-Cao *et al*., 2002; Matheu *et al*., 2004; Mendrysa *et al*., 2006). Moreover, the combined effects of modest and regulated increases in *p53* and *Ink4/Arf* result in a significantly elongated lifespan and delayed organismal aging (Matheu *et al*., 2007, 2009). These phenotypes are further extended in the presence of constitutive telomerase reverse transcriptase (Tomas-Loba *et al*., 2008). Additionally, the ablation of the Arf-p53 pathway, but not Ink4a, alleviates the premature aging of mice carrying hypomorphic mutant alleles of BubR1 mice with constitutively high levels of endogenous chromosome damage (Baker *et al*., 2008, 2013). This reveals a potent anti-aging activity of *Ink4/Arf* and p53 that is separable from their anticancer effects. However, the detailed anti-aging mechanisms exerted by *Ink4/Arf and p53* at the cellular level remain unresolved.

Neural stem cells (NSCs) are undifferentiated precursors that retain the ability to proliferate and self-renew, and they have the capacity to give rise to neurons and glia. The two major stem cell niches in the adult mammalian brain are the subventricular zone (SVZ) and the subgranular zone of the dentate gyrus (DG) of the hippocampus. There, quiescent NSCs proliferate slowly, but they can become activated and give rise to an intermediate population of fast-dividing transient amplifying progenitor cells, which rapidly differentiate to neuroblasts, which can also proliferate (Urban & Guillemot, 2014). Newly generated neuroblasts in the SVZ migrate in chains along the rostral migratory stream to become neurons in the olfactory bulb (OB), while neurons born in the DG, mature, and integrate into the local circuitry. It is well established that the activity of NSCs decreases with organismal aging contributing to the age-related impaired neurogenesis and neuronal differentiation. These reductions are paralleled by organismal cognitive decline and impaired behavioral performances (Fuentealba *et al*., 2012). In view of that, we consider of relevance to determine, in a direct manner and under normal physiological conditions, whether the concomitant increased dosage of *Ink4/Arf* and p53 might alleviate these impairments.

To characterize NSCs, we dissociated freshly isolated SVZ cells and established neurosphere cultures from young (1 month), adult (1 year), and old (2 years) *wt* and *s-Ink4/Arf/p53* mice. As expected, at least for the *wt* mice, we observed reduced neurosphere formation and self-renewal capacity [measured as secondary (2^ry^) neurospheres] in both genotypes with aging (Fig.1A,B). However, there were several notable differences between the two genotypes. There were significantly fewer 1^ry^ and 2^ry^ neurospheres derived from young *s-Ink4/Arf/p53* than from *wt* mice. In particular, young transgenic mice formed 61% and 71% 1^ry^ and 2^ry^ neurospheres relative to wt (Fig.1C,D). These differences were less pronounced and not statistically significant in 1-year-old mice, with similar numbers of both 1^ry^ (77%) and then 2^ry^ neurospheres (94%) being obtained from the two genotypes (Fig.1C,D). Moreover, this trend continued such that by 2 years, the number of 1^ry^ and 2^ry^ neurospheres obtained was now significantly higher from s-*Ink4/Arf/p53* than *wt* mice. Thus, transgenic mice developed, respectively, 160% and 146% 1^ry^ and 2^ry^ neurospheres relative to *wt* (Fig.1C,D). These results further confirm the relevant role that Ink4a and p53 play in the regulation of NSC proliferation and self-renewal (Molofsky *et al*., 2006, Meletis *et al*., 2006, Medrano *et al*., 2009) and suggest that an extra copy of normally regulated *Ink4/Arf* and *p53* delays the aging-associated decline of NSCs.

**Fig 1:**
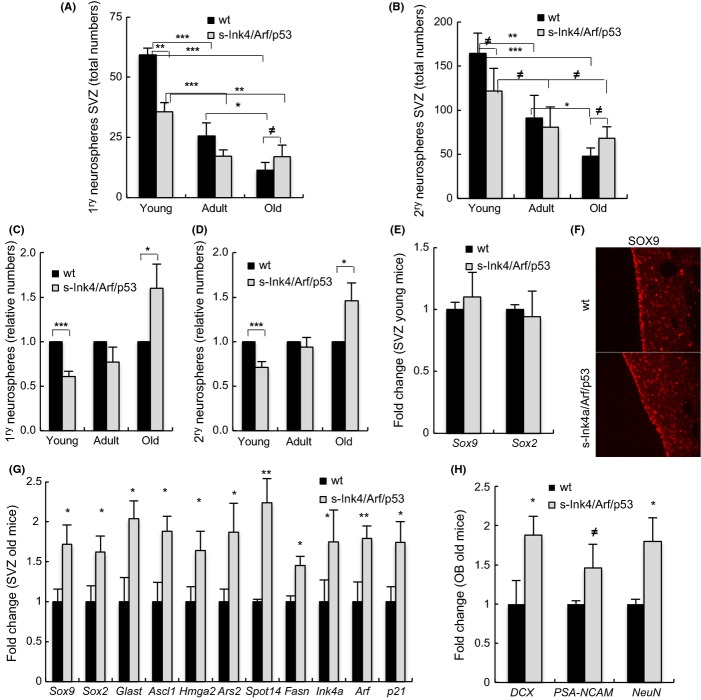
An extra copy of *Ink4/Arf* and p53 attenuates neural stem cells (NSCs) function decline in subventricular zone (SVZ) with aging. (A) Quantification of the number of 1^ry^ neurospheres formed from SVZ of 1-month (young), 12-month (adult), and 24- to 27- (old) month *wt* and s-*Ink4/Arf/p53* mice. Statistical differences were observed in both genotypes with aging comparing young with adult and old mice and also in 1-month s-*Ink4/Arf/p53* relative to *wt*. (B) The self-renewal potential of NSCs also declined with age but was statistically significant only in *wt* mice. Number of independent cultures *n* = 5 of each genotype from young and adult and *n* = 3 from old mice in both assays. (C, D) Quantification of the relative number of 1^ry^ and 2^ry^ neurospheres generated from transgenic SVZ relative to *wt*. (E) *Sox2* and *Sox9* mRNA levels in SVZ cells from 2-month-old mice (*n* = 2). (F) Representative image of SOX9 immunostaining from adult (9–12 months) mice (*n* = 3). (G) Quantification of NSC markers by qRT–PCR in SVZ from aged (24–30 months) mice. (H) Quantification of neuroblast and neuronal markers in olfactory bulbs from aged (24–30 months) mice of the indicated genotypes by qRT–PCR. Data are mean ± SEM, and the number of mice per genotype is at least *n* = 5 for both studies. The statistical comparisons were made using the Student’s *t*-test (asterisk ***P <* *0.005 and **P <* *0.01, *P <* *0.05, ^≠^*P *<* *0.1).

Next, we analyzed the NSCs population *in vivo*. Levels of SOX2 can be used to distinguish NSC from progenitors, with quiescent NSC showing high levels (Hutton & Pevny, 2011, Urban & Guillemot, 2014), while loss of *Sox2* leads to their differentiation (Favaro *et al*., 2009). High levels of SOX9 are also associated with NSCs (Scott *et al*., 2010). We checked the expression of these two genes in SVZ cells at different ages, finding that while there were no differences in their expression in young and adult *wt* and *s-Ink4/Arf/p53* animals (Fig.1E,F), aged transgenic mice presented significantly increased levels of *Sox2* and *Sox9* compared with *wt* mice (Fig.1G). This observation suggests that the NSC pool *in vivo* is similar in both genotypes in young mice, and is maintained in *s-Ink4/Arf/p53* in the elderly, further implying that an extra copy of normally regulated *Ink4/Arf* and *p53* delays the age-associated exhaustion of NSCs. To confirm this idea, the expression of additional quiescent NSC markers (including *Glast*, *Ars2*, or *Spot14*) was tested in old mice and we found that they were also elevated in the transgenic animals (Fig.1G). Similarly, the levels of *Ascl1* (activated progenitors) were higher in the SVZ niche of s-*Ink4/Arf/p53* (Fig.1G). The increased expression of these genes (1.5- to 2-fold higher) is within the range of overexpression expected for having an additional extra copy of regulated *Ink4/Arf* and *p53*. Indeed, the basal levels of *Ink4a*, *Arf*, and *p21*, used as a molecular readout of p53, were increased in cells from old transgenics in a similar range (Figs1G and 2C), endorsing that the transgenes are active and functional at advanced ages in the NSC pools. To test the functional activity of aged NSCs in SVZ, we determined the expression of *Doublecortin* (*DCX*) and *PSA-NCAM* (neuroblasts) and *NeuN* (neurons) in the OBs, noting that the expression of these genes was significantly elevated in aged *s-Ink4/Arf/p53* animals (Fig.1H). Together, our current data point out to a direct positive effect of the increased *Ink4/Arf* and *p53* dosage on NSC pools maintenance and NSC function with aging. Consequently, s-*Ink4/Arf/p53* mice present enhanced differentiation and sustain neurogenesis at late stages of their lifetime.

**Fig 2:**
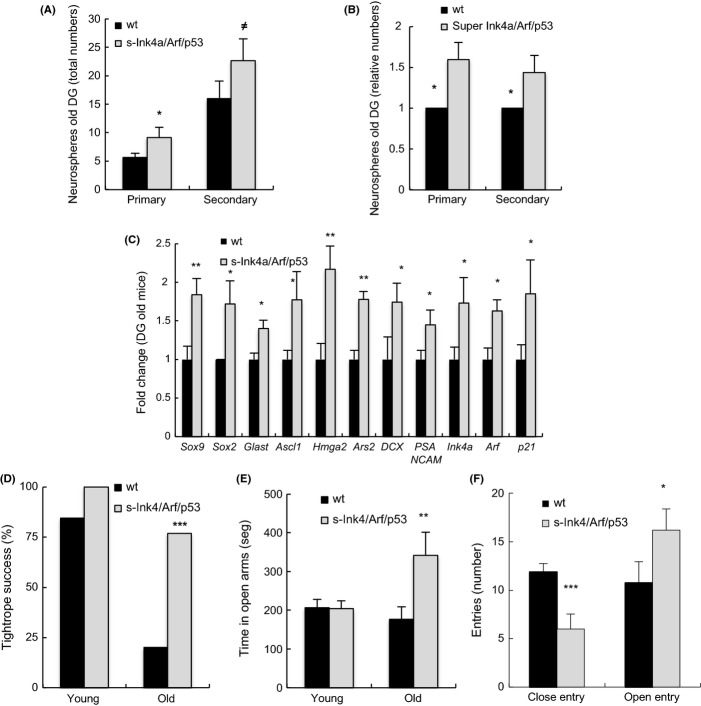
Aged s-*Ink4/Arf/p53* mice display improved dentate gyrus (DG) neural stem cell function and enhanced brain functional activity. (A) Quantification of the total and (B) relative number of 1^ry^ and 2^ry^ neurospheres formed from DG of more than 2-year-old *wt* and s-*Ink4/Arf/p53* mice (*n* = 3). (C) Determination of the mRNA levels of the indicated genes in DG from aged (24–30 months) mice. The number of mice per genotype is at least *n* = 4. Data are given as mean ± SEM, and statistical comparisons were made using the Student’s *t*-test. (D) Neuromuscular coordination was quantified as the percentage of mice that successfully passed the tightrope test. Number of mice is *wt* (young 13, aged 10) and s-*Ink4/Arf/p53* (young 6, aged 13). Statistical analysis was performed with Fisher’s exact test. (E) Total time spent in open arms, (F) and number of entries in open and closed arms in the elevated plus maze assay in the following number of mice: *wt* (young 10, aged 8) and s-*Ink4/Arf/p53* (young 4, aged 5). Young mice were between 6 and 10 months and aged mice ≥ 24 months. Data are presented as means ± SEM. Statistical analysis was performed with Student’s *t*-test compared with the aged *wt* group (asterisk ***P <* *0.005 and **P <* *0.01, *P <* *0.05, ^≠^*P *<* *0.1).

To further characterize NSC populations in old transgenic mice, we studied their activity in the DG. Similar to the SVZ, aged s-*Ink4/Arf/p53* DG cells generated increased number of 1^ry^ and 2^ry^ neurospheres compared with *wt* (Fig.2A,B). Furthermore, the expression of *Sox2, Sox9*, *Glast*, *Ascl1*, *Hmga2*, and *Ars2* was elevated in the DG niche *in vivo* (Fig.2C). Finally, the expression of *DCX* and *PSA-NCAM* was also increased in more than 2-year-old *s-Ink4/Arf/p53* mice (Fig.2C). Collectively, these data confirm that an extra copy of combined *Ink4a/Arf* and *p53* attenuates both the exhaustion in NSCs activity and the subsequent decline in neurogenesis in the two major neurogenic regions of the adult mouse brain.

Finally, we investigated the behavioral consequences of the *Ink4/Arf* and *p53* extra copy with aging. For this, transgenic mice were subject to two different functional tests: tightrope and elevated plus maze, performances in which are well established to worsen with aging and have been linked to p53 activity (Scrable *et al*., 2009; Kim & Wong, 2012). In both tests, there were no differences between the two genotypes in young mice (Fig.2D,E). However, the performance of aged transgenics in the tightrope test was significantly improved. Indeed, when comparing mice older than 2 years, 75% of s-*Ink4/Arf/p53* animals successfully passed this test, compared with only 20% of the *wt* group (Fig.2D). In the elevated plus maze test, aged s-*Ink4/Arf/p53* mice spent a significantly longer time (Fig.2E) and showed an improved tendency to explore open arms, compared with the *wt* group (Fig.2F). Furthermore, the time and percentages of entries (Fig.2F) in the closed arms were significantly lower. Therefore, an extra copy of *Ink4/Arf* locus and *p53* ameliorates age-associated loss of neuromuscular coordination, retains cognitive performance and locomotor activity, and decreases anxiety-related stress.

In summary, *s-Ink4/Arf/p53* animals show an overall greater number of NSCs within the SVZ and DG niches with aging compared with controls. This is also reflected by the enhanced stem cell frequency and self-renewal potential seen *in vitro*. In line with this result, skin epithelial stem cells derived from s-*Ink4/Arf/p53* mice with more than 2.5 years old also display increased clonogenic potential compared with controls (Tomas-Loba *et al*., 2008). Moreover, they express significantly higher levels of several markers of quiescent NSCs as well as fast-dividing progenitors *in vivo*, together indicating that an extra copy of regulated *Ink4a/Arf and* p53 delays age-associated decline of NSCs pools. The relative increase in *Sox2* and *Sox9* expression only at later stages of the lifetime of the mice suggests that *Ink4/Arf* and *p53* modulate NSC activity contributing to their quiescence and long-term maintenance. Given that mice overexpressing specifically Ink4a only show a minimal modification in SVZ proliferation in old mice and without differences in NSC numbers or their self-renewal (Molofsky *et al*., 2006), the effects of the s-*Ink4/Arf/p53* transgenes on NSCs might be explained mainly by the activity of the Arf-p53 pathway. Consistent with this idea, ablation of p53 or its target p21 results in loss of quiescence and depletion of long-term neural stem cells at advanced ages (Kippin *et al*., 2005; Meletis *et al*., 2006), and short-lived mice with hyperactivated p53 present premature exhaustion of NSCs in the adult SVZ (Medrano *et al*., 2009). Mechanistically, *Arf/p53* might protect stem cell exhaustion, through its ability to decrease proliferation rates and its anti-oxidant activity (Matheu *et al*., 2007).

An extra copy of regulated Ink4/Arf and p53 also delays age-associated decline of neurogenesis and neuronal differentiation. This is shown by the elevated levels of both markers of newly generated neuroblasts (DCX and PSA-NCAM) and neurons (NeuN) within the OBs and DG of aged s-Ink4/Arf/p53 mice. In agreement with higher stem cell function, aged *s-Ink4/Arf/p53* mice display increased ability to hair regrow (Matheu *et al*., 2007). Finally, we show that the delay in NSCs and neurogenesis reduction observed with aging in the transgenic mice are paralleled by improved behavioral performances, indicative of enhanced brain functional activity in transgenic mice. These experiments, particularly the tightrope test, also postulate that transgenic mice present greater muscle function. In the case of the elevated plus maze experiment, our results add novel information to the link between tumor suppression, stem cell biology and anxiety. While deregulated p53-induced NSCs exhaustion has been proposed to contribute to anxiety disorders (Scrable *et al*., 2009), normally regulated p53 delays NSCs aging along with a reduction in anxiety-related behaviors. In conclusion, our results demonstrate that *Arf/p53* exerts anti-aging activity by, at least in part, protecting stem cell exhaustion, providing a rationale not only for the extended lifespan but also for the health span of these mice.

## References

[b1] Baker DJ, Perez-Terzic C, Jin F, Pitel K, Niederlander NJ, Jeganathan K, Yamada S, Reyes S, Rowe L, Hiddinga HJ, Eberhardt NL, Terzic A, van Deursen JM (2008). Opposing roles for p16Ink4a and p19Arf in senescence and ageing caused by BubR1 insufficiency. Nat. Cell Biol.

[b2] Baker DJ, Weaver RL, van Deursen JM (2013). p21 both attenuates and drives senescence and aging in BubR1 progeroid mice. Cell Rep.

[b3] Dumble M, Moore L, Chambers SM, Geiger H, Van Zant G, Goodell MA, Donehower LA (2007). The impact of altered p53 dosage on hematopoietic stem cell dynamics during aging. Blood.

[b4] Favaro R, Valotta M, Ferri AL, Latorre E, Mariani J, Giachino C, Lancini C, Tosetti V, Ottolenghi S, Taylor V, Nicolis SK (2009). Hippocampal development and neural stem cell maintenance require Sox2-dependent regulation of Shh. Nat. Neurosci.

[b5] Fuentealba LC, Obernier K, Alvarez-Buylla A (2012). Adult neural stem cells bridge their niche. Cell Stem Cell.

[b6] Garcia-Cao I, Garcia-Cao M, Martin-Caballero J, Criado LM, Klatt P, Flores JM, Weill JC, Blasco MA, Serrano M (2002). “Super p53” mice exhibit enhanced DNA damage response, are tumor resistant and age normally. EMBO J.

[b7] Gatza CE, Dumble M, Kittrell F, Edwards DG, Dearth RK, Lee AV, Xu J, Medina D, Donehower LA (2008). Altered mammary gland development in the p53 + /m mouse, a model of accelerated aging. Dev. Biol.

[b8] Hutton SR, Pevny LH (2011). SOX2 expression levels distinguish between neural progenitor populations of the developing dorsal telencephalon. Dev. Biol.

[b9] Kim J, Wong PK (2012). Targeting p38 mitogen-activated protein kinase signaling restores subventricular zone neural stem cells and corrects neuromotor deficits in Atm knockout mouse. Stem Cells Transl. Med.

[b10] Kippin TE, Martens DJ, van der Kooy D (2005). p21 loss compromises the relative quiescence of forebrain stem cell proliferation leading to exhaustion of their proliferation capacity. Genes Dev.

[b11] Maier B, Gluba W, Bernier B, Turner T, Mohammad K, Guise T, Sutherland A, Thorner M, Scrable H (2004). Modulation of mammalian life span by the short isoform of p53. Genes Dev.

[b12] Matheu A, Pantoja C, Efeyan A, Criado LM, Martin-Caballero J, Flores JM, Klatt P, Serrano M (2004). Increased gene dosage of Ink4a/Arf results in cancer resistance and normal aging. Genes Dev.

[b13] Matheu A, Maraver A, Klatt P, Flores I, Garcia-Cao I, Borras C, Flores JM, Vina J, Blasco MA, Serrano M (2007). Delayed ageing through damage protection by the Arf/p53 pathway. Nature.

[b14] Matheu A, Maraver A, Collado M, Garcia-Cao I, Canamero M, Borras C, Flores JM, Klatt P, Vina J, Serrano M (2009). Anti-aging activity of the Ink4/Arf locus. Aging Cell.

[b15] Medrano S, Burns-Cusato M, Atienza MB, Rahimi D, Scrable H (2009). Regenerative capacity of neural precursors in the adult mammalian brain is under the control of p53. Neurobiol. Aging.

[b16] Meletis K, Wirta V, Hede SM, Nister M, Lundeberg J, Frisen J (2006). p53 suppresses the self-renewal of adult neural stem cells. Development.

[b17] Mendrysa SM, O’Leary KA, McElwee MK, Michalowski J, Eisenman RN, Powell DA, Perry ME (2006). Tumor suppression and normal aging in mice with constitutively high p53 activity. Genes Dev.

[b18] Molofsky AV, Slutsky SG, Joseph NM, He S, Pardal R, Krishnamurthy J, Sharpless NE, Morrison SJ (2006). Increasing p16INK4a expression decreases forebrain progenitors and neurogenesis during ageing. Nature.

[b19] Scott CE, Wynn SL, Sesay A, Cruz C, Cheung M, Gomez Gaviro MV, Booth S, Gao B, Cheah KS, Lovell-Badge R (2010). SOX9 induces and maintains neural stem cells. Nat. Neurosci.

[b20] Scrable H, Burns-Cusato M, Medrano S (2009). Anxiety and the aging brain: stressed out over p53?. Biochim. Biophys. Acta.

[b21] Tomas-Loba A, Flores I, Fernandez-Marcos PJ, Cayuela ML, Maraver A, Tejera A, Borras C, Matheu A, Klatt P, Flores JM, Viña J, Serrano M, Blasco MA (2008). Telomerase reverse transcriptase delays aging in cancer-resistant mice. Cell.

[b22] Tyner SD, Venkatachalam S, Choi J, Jones S, Ghebranious N, Igelmann H, Lu X, Soron G, Cooper B, Brayton C, Park SH, Thompson T, Karsenty G, Bradley A, Donehower LA (2002). p53 mutant mice that display early ageing-associated phenotypes. Nature.

[b23] Urban N, Guillemot F (2014). Neurogenesis in the embryonic and adult brain: same regulators, different roles. Front. Cell. Neurosci.

